# Engineering of Shallow Layers of Nitrogen Vacancy Colour Centres in Diamond Using Plasma Immersion Ion Implantation

**DOI:** 10.1038/s41598-019-42323-6

**Published:** 2019-04-10

**Authors:** Fahad Alghannam, Philip Hemmer

**Affiliations:** 1 National Center for Laser and Optoelectronics Technologies, KACST, Riyadh, Saudi Arabia; 2 Centre for Quantum Optics and Quantum Infromatics, KACST, Riyadh, Saudi Arabia; 3 Electrical and Computer Engineering, Texas A&M University, College Station, TX 77843-3128 USA; 4 Zavoisky Physical-Technical Institute, Federal Research Centre “Kazan Scientific Centre of RAS”, Kazan, Russia

## Abstract

Sensing nano-scale magnetic field sources is at the heart of many applications in nano-science and biology. Given its very small size and high magnetic sensitivity, the nitrogen vacancy (NV) colour centre in diamond is one of the leading candidates for such applications. However, issues regarding the stability and performance of the NV centre near the diamond’s surface are the major obstacle in the practical realization of theses sensors. So far, conventional implantation and growth techniques did not produce practical and/or repeatable solutions to this problem. In this report, we show first results of shallow layers of NVs created using plasma immersion ion implantation (PIII). We show, using Forster Resonance Energy Transfer (FRET), that most NVs are within 3.6 nm from the diamond’s surface. Despite the relatively low quality of the diamond substrates used and the simplicity of our PIII system, we have an estimated magnetic sensitivity of around 2.29 *μT*/$\sqrt{Hz\cdot \mu {m}^{-2}}$.

## Introduction

Despite the major breakthroughs in magnetometry in the last three decades or so, currently available magnetometers fall short in many sensing applications in nano-science and biology. In particular, most magnetometers are too big or too slow for applications which involve dynamical nanoscale magnetic field sources. Tracking a single paramagnetic atom, or detection of the action potential of a single neuron are examples of such applications. Some of the magnetometry technologies with highest magnetic sensitivity include; vapor cells^1,2^, superconducting quantum interference devices **(**SQUID)^3,4^, fibre optic magnetometers^5^, magneto optics^6^, Microelectromechanical systems (MEMS), and magnetic resonance force microscopy (MRFM)^7^. Given the obvious trade-off between size and magnetic sensitivity, when miniaturization of magnetometers is attempted, the loss in magnetic sensitivity limits the usefulness of the magnetometer.

In diamond, the NV colour centre has been utilized as an atomic size magnetic sensor^8,9^. The localization of the NV made it a leading candidate for sensing nanoscale magnetic field sources. The NV is a deep defect in diamond consisting of a substitutional nitrogen atom next to a vacancy in the diamond lattice. Very high magnetic sensitivity was demonstrated for NVs in bulk diamond^10^. Also, a single NV was used to detect an individual proton on the diamond surface using quantum reporters^11^. Furthermore, optical detection of a single neuron action potential was demonstrated with a 13-*μm* thick layer of NVs grown on bulk diamond^12^. These examples show the impressive capabilities and potential of the NV as a magnetometer.

The NV has two stable fluorescent charge states; the neutrally charged *NV*^0^, and the negatively charged *NV*^−^. Both charge states are paramagnetic, however, only *NV*^−^ shows optical detection of magnetic resonance (ODMR). The spin state of *NV*^−^ can be detected optically, and in room temperature, which is the main advantage of *NV*^−^ over *NV*^0^ and other optically active paramagnetic defects in diamond. There are many theoretical studies attempting to understand different aspects of the NV color center^13^. Since we want to focus on the advantages of PIII implantation in creating shallow layers of *NV*^−^ in bulk diamond, we will only briefly explain the physics of the NV.

**Figure 1:**
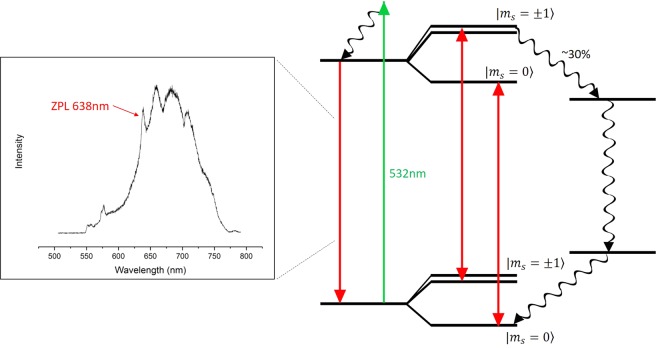
A simple energy level diagram for the *NV*^−^ colour centre including a typical emission spectrum.

Since we are using *NV*^−^ as the magnetic sensor here, we will be referring to it as NV throughout this report. The NV’s ground state is a spin triplet with a zero-field splitting of 2.87 GHz between $$|{m}_{s}=0 > $$ and $$|{m}_{s}=\pm \,1 > $$ states. It is usually excited with green laser to another spin triplet state and has a broad-band emission in the range 630 nm–800 nm with a zero-phonon line at 638 nm. Figure (1) shows the energy diagram and optical spectrum for the NV centre. As mentioned earlier, the most attractive feature of the NV is that it allows ODMR. ODMR is possible since the NV’s brightness depends on the spin state. When an NV in $$|{m}_{s}=0 > $$ state is optically excited it, almost always, relaxes by emitting a red photon. In contrast, when the NV is in $$|{m}_{s}=\pm \,1 > $$ states, there is approximately 30% chance it will relax non-radiatively through singlet states^8^.

Despite the high magnetic sensitivity of NVs and their very small size, measuring nano-scale magnetic field sources using NVs is still a challenge. Next to high magnetic sensitivity, proximity to the field source is essential for such sensors. Therefore, a high quality shallow layer of NVs is desirable. When using conventional implantation or growth techniques, shallow NVs (<5 nm from the surface) are mostly stable in the neutrally charged state^14,15^. Even when a shallow NV maintains its charge, it usually has very short relaxation time leading to low magnetic sensitivity^16–22^. In this report, we show that the plasma immersion ion implantation (PIII) technique applied to type Ib diamond can create a dense shallow layer of NVs that is optimal for nano-scale magnetic field sensing applications.

In conventional implantation, accelerated ions penetrate the diamond lattice leaving a trail of damage^23^. This damage accumulates on the shallower layers preventing NVs from forming. To overcome this problem, good quality, very shallow NVs were created by implanting nitrogen deep into the diamond first, then etching the damaged layers to get the NV closer to the surface^24^. However, implanting and etching in two different steps creates a major repeatability issue since the implantation energy and the etching parameters need to be very accurately measured and controlled.

**Figure 2:**
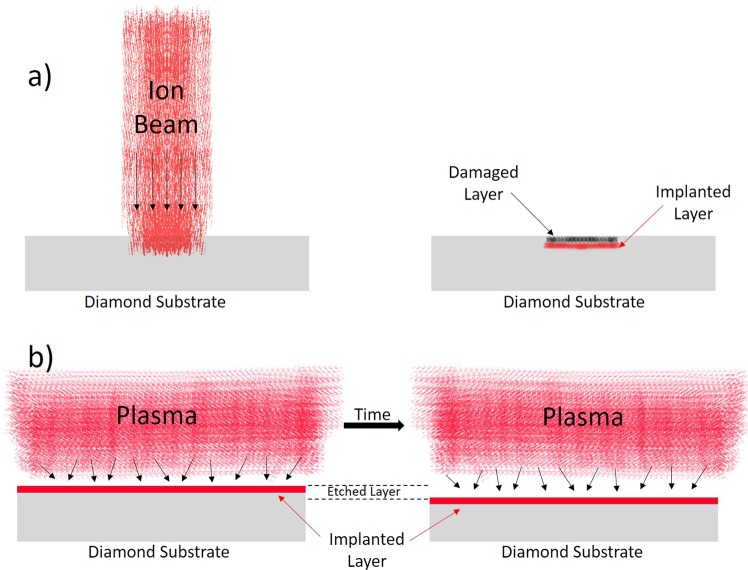
An illustration of the difference between conventional implantation (**a**) and PIII (**b**). In PIII the damaged layers are etched continuously resulting in shallower implanted layers.

PIII is an implantation technique where the substrate is immersed in a plasma of the implantation gas. In general, conventional implantation provides more control over implantation energy, however, for our application PIII is more suitable. PIII utilizes simultaneous implantation and etching resulting in a shallow good quality implantation layer. Figure (2) shows an illustration of the difference between PIII and conventional implantation. In PIII, the implantation process quickly reaches a steady state where the implanted layer properties become independent from implantation/etching time. Hence, repeatability is built in.

## Experimental Setup

**Figure 3:**
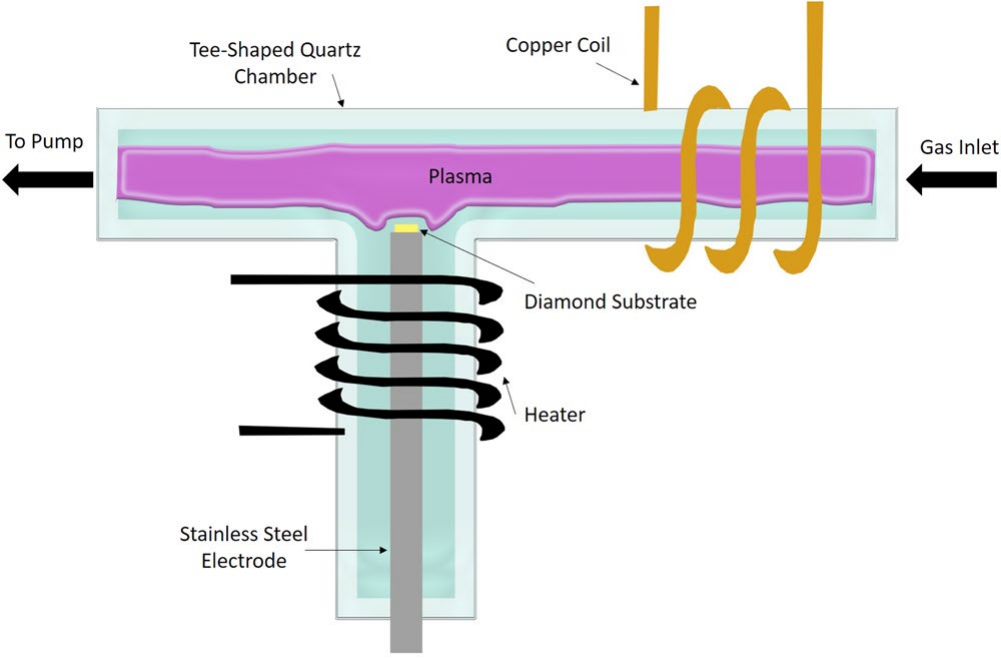
Schematic of our compact PIII implanter.

In this work, we use a home built PIII system, Fig. (3). Our system consists of an implantation chamber, a vacuum system, a gas inlet equipped with a mass flow controller (MFC), a pusler circuit connected to a high voltage dc supply, and an RF source with matching network that drives a copper coil. The implantation chamber is a “T” shaped quartz tube. Nitrogen gas flows through the MFC from one end of the tube to the other end, where the vacuum pumps are attached. A stainless-steel electrode holding the diamond substrate is inserted from the side and the chamber is sealed. To generate plasma, a coil made of copper tube is looped around the implantation chamber as shown in Fig. (3). This copper coil is attached to Advanced Energy’s VM1500AW impedance matching network, which is driven by a 13.56 MHz RF power supply model R301 from Seren. Cooling water is running through the coil during operation.

First the chamber is pumped to high vacuum. Then, 50 sccm of nitrogen gas flows though the tube. After that, around 200 W of RF power is sent through the coil to generate plasma. To drive the nitrogen ions from the plasma into the diamond substrate, a high negative dc voltage is needed. However, if the voltage is kept on for a long time the plasma will quench. Therefore, a home built pulser circuit is connected between the power supply and the electrode. The implantation voltage ranges from −4 kV to −8 kV and the implantation frequency is 250 Hz with %10 duty cycle. To investigate the effect of implantation temperature, a high resistance heating wire was looped around the implantation chamber near the diamond holder.

To characterize the NV layers, we use a home built confocal microscope. This microscope is equipped with a 532 nm green laser. First, the laser beam goes through a single mode optical fibre which works as a spatial filter. Then, the beam is guided with two Galvano mirrors to scan the sample in XY. The Galvano scanner is imaged to the back focal plane of a high NA objective lens. The objective focuses the laser beam onto the sample. The fluorescence is collected using the same objective. It is then focused through a 150 um pinhole using a 30 cm lens. Finally, the signal is divided with a 50/50 beam splitter where one leg is focused on an APD and the other on a home-built spectrometer. To add Z scanning capabilities, the objective lens is mounted on an open loop piezo stage.

**Figure 4:**
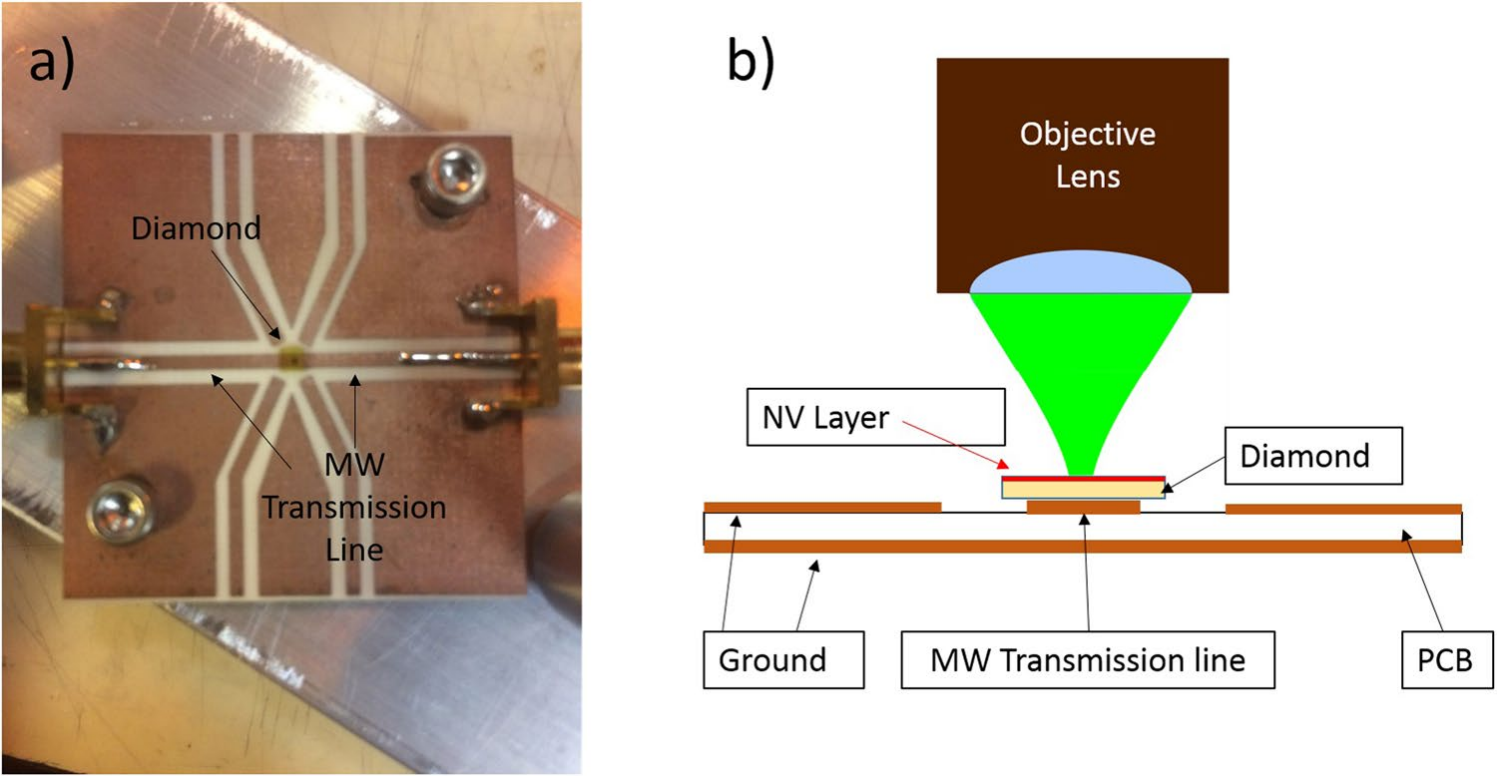
MW excitation apparatus. (**a**) a picture of a PCB board that was used to deliver MW excitation to the samples. (**b**) a drawing illustrating how diamond substrates were mounted to generate ODMR spectra.

In order to generate ODMR spectra, the diamonds were mounted on a printed circuit board (PCB) designed to deliver the microwave (MW) excitation to the NVs. Figure (4a), shows a picture of one of the PCB boards used in this work. Figure (4b), illustrates how the diamond was mounted in the confocal microscope. The MW signal is generated using an Analog device ADF4351 frequency synthesizer then amplified with mini-circuits ZHL-16W-43 MW amplifier. To supress low frequency noise, mostly from the laser and mechanical vibrations, a digital equivalent of Lock-in amplifier (LIA) technique was employed for some of the measurements. Briefly, we modulated the MW field using ZASW-2–50DR MW switch and then demodulated the optical signal collected by the APD in software.

## Results and Discussion

To test our implantation scheme, we used type Ib diamonds from Element Six and Sumitomo. Typically, a type Ib diamond contains around 200 ppm nitrogen concentration. Even though, it contains many nitrogen atoms, there are almost no NVs and the diamond is not fluorescent under green excitation. For our application, type Ib is suitable for two reasons. First, a high density of NVs can be created with lower implantation dose since the diamond lattice has nitrogen in it to begin with. Second, and more importantly, the diamond is “n-doped” which will make the NV charge state more stable even for very shallow NVs. It is important to add that upon testing our implanter on an ultrapure diamond, NVs were created, however, they were very dim and had low quality.

**Figure 5:**
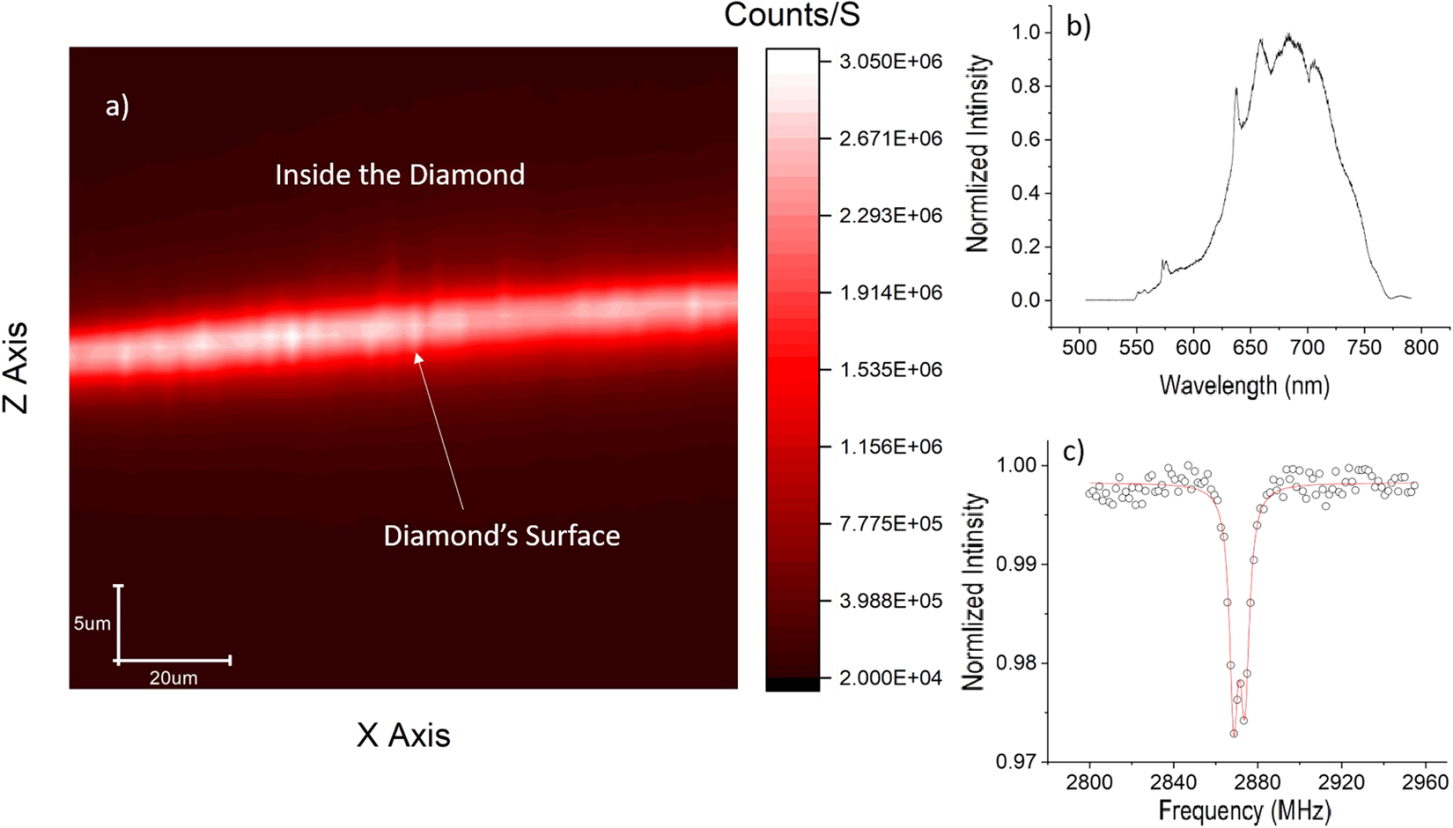
Shallow Implantation Results. (**a**) a confocal XZ scan showing a bright surface of a type Ib diamond implanted with PIII. (**b**) Optical spectrum of the shallow layer in (a) which matches that of the NV. (**c**) ODMR spectrum of the layer shown in (a).

Figure (5a) shows a confocal XZ scan of a shallow layer of NVs created on a type Ib diamond from element6 using our PIII implanter. The implantation was performed at room temperature and the implantation voltage was −4 kV. After the implantation, the sample was investigated under the confocal microscope but no NVs were found. This is in accordance with typical room temperature implantation since implantation results in nitrogen defects and vacancies but not necessarily NVs. Post implantation annealing is necessary to allow diffusion of vacancies until they are trapped by substitutional nitrogen, thus creating the NV centre. For this sample the NV layer was found after annealing at 890 C for 2.75 hours in vacuum. From the XZ scan we can see an intense fluorescence from a layer on the diamond surface. From simulation studies on ion implantation in diamond^25,26^, the estimated thickness of this layer will be 10 s of nanometres at most. This is well below the axial resolution limit of a conventional optical confocal microscope which is around 0.5 micron at best. Figure (5b) shows the optical spectrum from this layer which matches that of the NV centre. Figure (5c) shows the ODMR spectrum of this NV ensemble.

Since the thickness and depth of these NV layers are much smaller than the confocal resolution limit, we need to employ a different method to estimate these quantities. For this we utilize FRET. FRET is a near field phenomena where energy transfers non-radiatively from an emitter to an acceptor atoms or molecules. FRET efficiency can be understood as the probability of an emitter photon to be absorbed by a nearby acceptor through FRET as opposed to the photon being detected in the far field. FRET efficiency is given by:

$$E=\frac{{{R}_{0}}^{6}}{{{R}_{0}}^{6}+{R}^{6}}$$

Where *R*_0_ is the FRET radius and *R* is the distance between the emitter and the acceptor. The sharp decay of the efficiency with distance makes FRET suitable for our purpose. We can effectively say that the emitters fluorescence will be quenched if the acceptor is closer than *R*_0_ and will not be affected otherwise. For this experiment we use the NV as the emitter and the black hole quencher BHQ3 as the acceptor. Quenching NV fluorescence with BHQ3 has been demonstrated successfully with calculated FRET radius ${R}_{0}=3.6\,nm$ ^27^.

**Figure 6:**
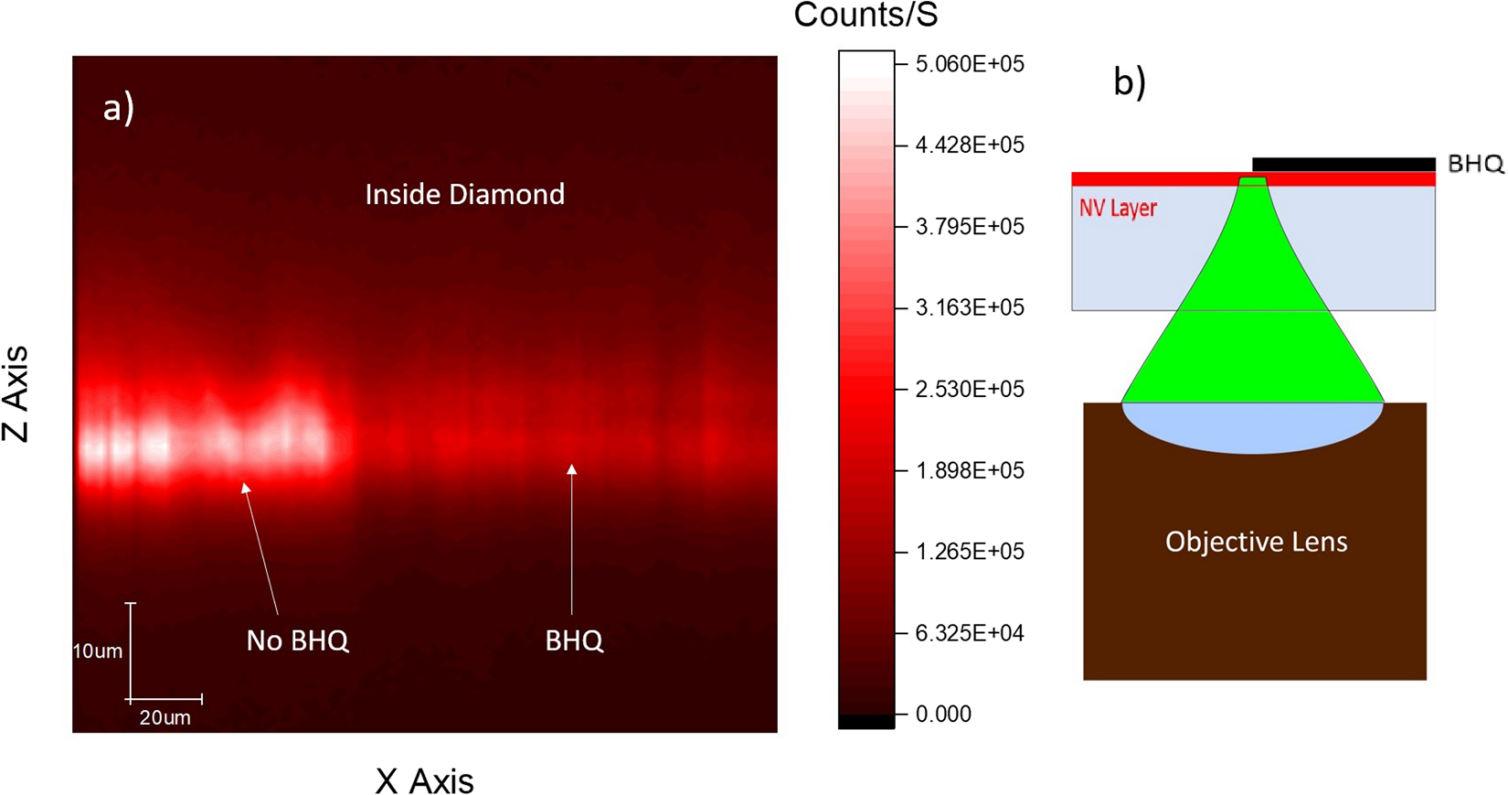
NVs’ depth estimation experiment using FRET (**a**) a confocal XZ scan which illustrates the effect of BHQ3 quenching. The right side of the diamond is covered with BHQ3 while the left side is not. (**b**) Illustration of the experiment in (a). The NV layer was illuminated, and the fluorescence was collected, through the diamond.

In this experiment we implanted an HPHT type Ib diamond from Sumitomo with our PIII implanter. To see the effect of FRET we masked half the diamond face with scotch tape. Then, a drop of BHQ3 was put on the diamond surface and left to dry. Finally, the tape was removed, and the sample was investigated under the confocal microscope. To make sure BHQ3 does not block the laser or the fluorescence, the NV layer was illuminated through the diamond as shown in Fig. (6b). Figure (6a) shows an XZ scan where NVs under BHQ3 suffer from significant quenching. From this result we can say that most NVs are indeed closer than 3.6 nm from the diamond surface. It is important to add that; the same procedure was applied to a shallow layer of NVs grown with chemical vapor deposition (CVD) on an ultrapure diamond substrate and no quenching was observed.

So far, we have shown the ability of PIII implantation in the creation of very shallow layer of NVs which address the proximity requirement for nano-scale magnetic field sensing. The other requirement is good magnetic sensitivity. The magnetic sensitivity of an NV ensemble is directly related to the width and contrast of the ODMR signal. The sensitivity also improves with number of NVs in the interrogation volume.

**Figure 7:**
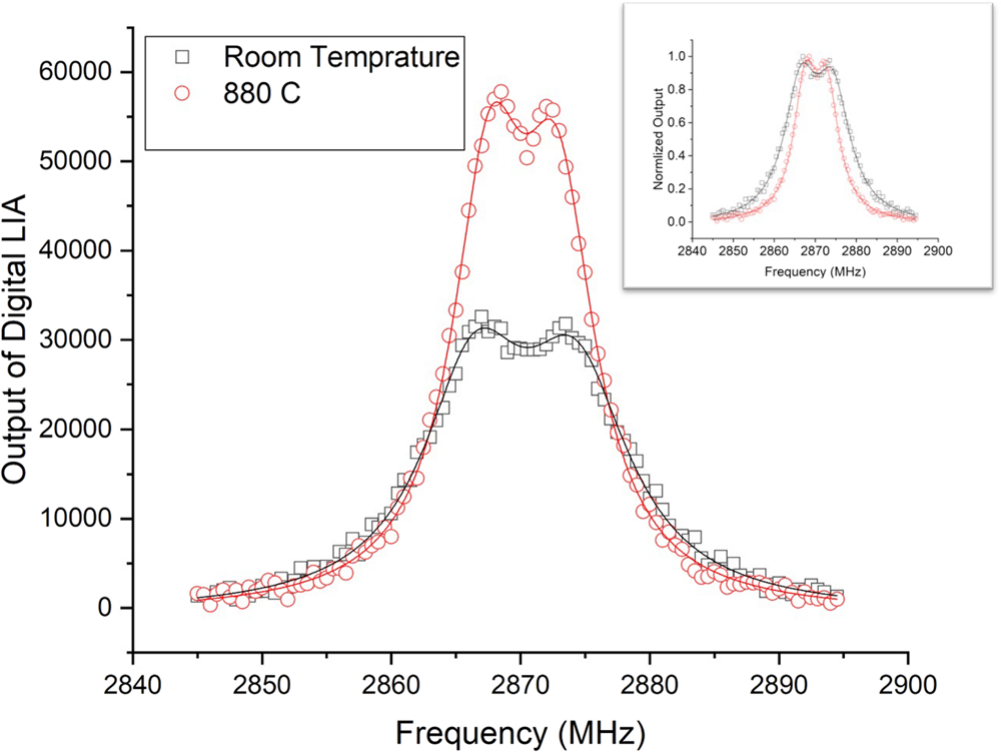
A comparison between the ODMR spectrum of two similar type Ib diamonds from Sumitomo implanted with PIII at room temperature (black) and at 880 C (red). Both spectra were taken at room temperature. The two spectra are normalized in the inset.

To improve the NV layer quality, we preformed high temperature implantation. Figure (7) shows a comparison between the ODMR spectrum of shallow NVs implanted on two similar type Ib Sumitomo diamonds at room temperature and at 880 C. High temperature implantation improved the width and contrast of the ODMR peak. During high temperature implantation, vacancies are constantly diffusing. This suppresses creation of long vacancy chains which usually occur during post implantation annealing for high-dose room temperature implantation^23^. In Fig. (7), the signal to noise ratio of the ODMR spectra were enhanced using a digital form of Lock-in amplifier (LIA). More details about this technique can be found under “Methods”.

**Figure 8:**
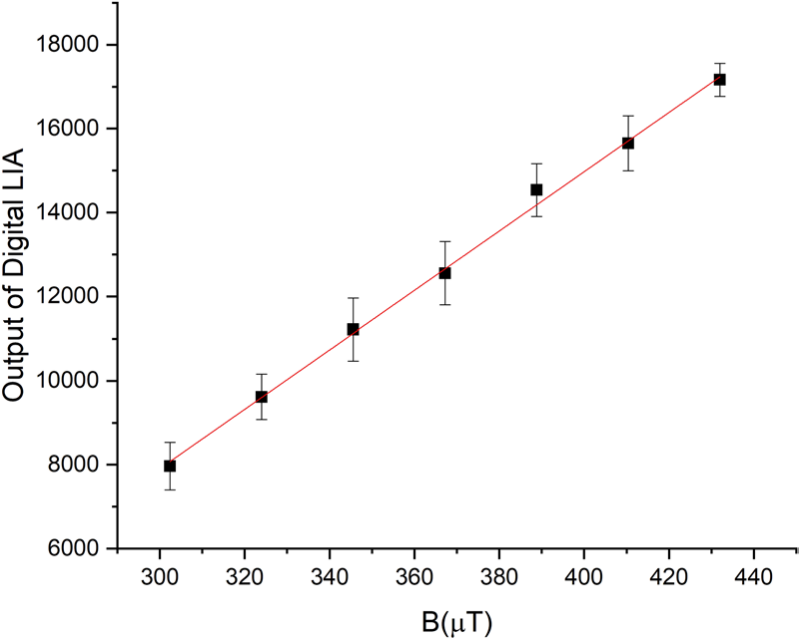
Direct measurement of magnetic sensitivity of a shallow layer of NVs created with PIII implantation. The magnetic field was along the <001> axis. Each measurement was repeated 10 times and the average is plotted. The error bars represent the standard deviation of the measurements.

To estimate the magnetic sensing performance of our shallow NV layers, we directly measured the magnetic sensitivity of a shallow NV layer which was implanted using our PIII system on a type Ib element6 diamond. The diamond was implanted at 800C with implantation voltage of −4 kV. The magnetic field was roughly aligned along the <001> axis. Figure (8) shows the brightness of the layer’s fluorescence as a function of the magnetic field. The frequency synthesizer was locked on 2860 MHz and chopped at 1 kHz. The measurement was repeated 10 times at each magnetic field. The error bars in Fig. (8) represent the standard deviation of the measurements. This particular shallow layer has a sensitivity around $8.6\,\mu T/\sqrt{Hz}$. A more useful quantity is the magnetic sensitivity per unit area. Since we have a uniform shallow layer, we have the option to increase the magnetic sensitivity by increasing the interrogation area, at the expense of spatial resolution. The magnetic sensitivity per unit area for the sample in Fig. (8) is $2.29\,\mu T/\sqrt{Hz\cdot \mu {m}^{-2}}$. If we assume the layer thickness to be around 3 nm, i.e. 2 nm to 5 nm from the diamond surface, which is reasonable given the FRET results, we can calculate the magnetic sensitivity per unit volume for this layer to be $125\,nT/\sqrt{Hz\cdot \mu {m}^{-3}}$.

In conventional implantation, the dose is easily calculated from the ion beam flux and the implantation time. However, it is not that simple when it comes to PIII. One can try to measure the current running through the electrode but that is a poor parameter especially when implanting insulator substrates like diamond. Furthermore, at the steady state, the dose does not depend on the implantation time. At the steady state the dose depends on the implantation rate, implantation depth, and etching rate. These parameters can be controlled by controlling the implantation energy, changing the gas mixture, or changing the electrode design. So far, we have not attempted to manipulate any of these parameters since our compact and economical PIII system does not support these manipulations. Even though we might not know how much nitrogen was implanted, we can estimate the density of NVs in the implanted layer from their areal brightness. In a diffraction limited laser spot, we have 50-90 *NVs* depending on the sample.

**Figure 9:**
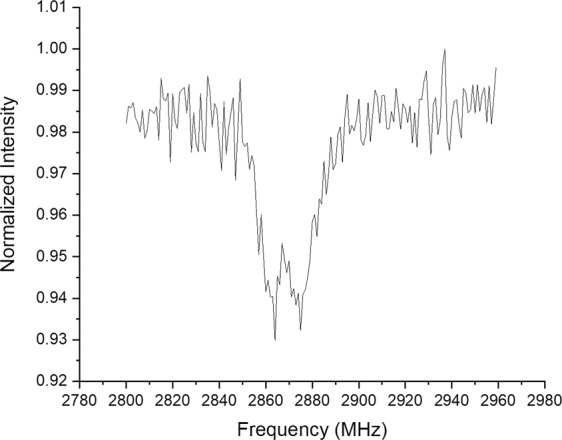
An ODMR spectrum of one of our shallow layers generated using 636 nm optical excitation.

One more advantage of using “n-doped” diamond in this work is that NVs hosted in such diamonds can be efficiently excited with red laser. Replacing, the phototoxic, green excitation with red is essential for many bio sensing applications^28^. Normally, red excitation would deplete the NV fluorescence and sends it to a dark state^29–31^. However, since we are using “n-doped” diamond substrates, we are able to generate ODMR spectra, and preform optical magnetic sensing, with our NVs with “bio-safe” red lasers. The physics behind this phenomenon is well explained by^32^. Figure (9) shows an ODMR spectrum generated with 636 nm excitation laser.

## Conclusion

In this report we show an innovative method for creating shallow layers of NVs suitable for sensing nano-scale magnetic field sources. Using a low-cost compact custom-built PIII implanter, we were able to create very shallow layer of NVs in type Ib diamonds. Using the FRET interaction between NVs and BHQ3 molecules we showed that most NVs are indeed within 3.6 nm (FRET radius) from the surface. High temperature PIII implantation resulted in a shallow NV layer with higher magnetic sensitivity compared to room temperature implantation. We can estimate that our technique gives a magnetic sensitivity per unit area around $2.29\,\mu T/\sqrt{Hz\cdot \mu {m}^{-2}}$. Finally, we show that our shallow layers are more suitable for biosensing applications, compared to NVs in ultra-pure diamonds, since it can be optically excited with red light.

It is important to note that the diamond substrates used in this work are low-cost, marginally polished, diamond plates that are usually used for thermal sink applications. We expect the magnetic sensitivity to improve significantly when using higher quality diamonds with superior polishing. We hope that our work will open the door for more investigation of PIII implantation in diamond. Our primitive PIII system with the most basic electrode design leaves much room for future optimization.

## Methods

### Magnetic Field Sensing with NV ensembles and Magnetic Sensitivity

**Figure 10:**
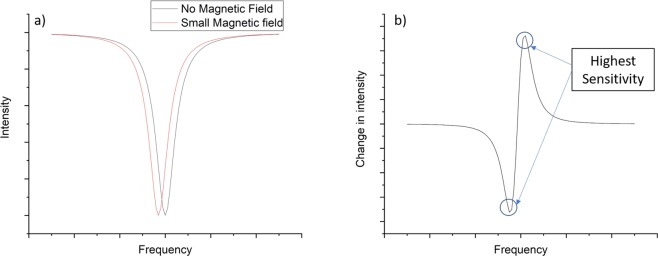
Illustration of CW magnetic sensitivity. (**a**) Shift of an ideal ODMR peak due to a small magnetic field. (**b**) Change in fluorescence intensity due to a small magnetic field (red curve – black curve).

Mainly, there are three categories of NV-based magnetometry experiments; continuous wave (CW), pulsed, and relaxomentery. In this report we have only demonstrated CW magnetometry. Figure (10a), shows the effect of a small magnetic field on an ideal ODMR spectrum. A magnetic field B along the quantization axis of the NV shifts the ODMR peak by *γB*, where $\gamma =28\,GHz/T$ is the NV’s magnetic field coupling coefficient^33–35^. The value of the magnetic field can be calculated by fitting the ODMR data and finding the peak’s location. More practically, however, we can simply monitor the NVs’ fluorescence at the MW frequency corresponding to the maximum change in the NVs’ fluorescence. Figure (10b) shows the points of maximum change which correspond to highest sensitivity.

**Figure 11:**
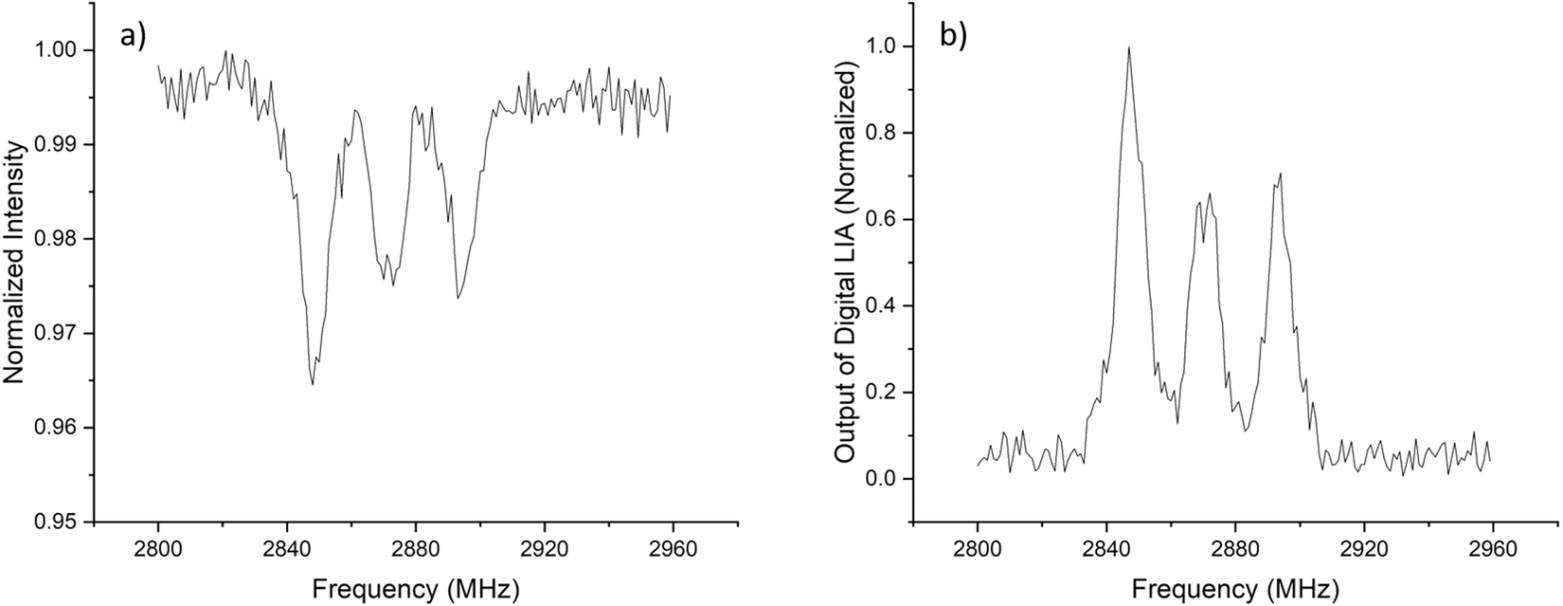
Illustration of digital LIA noise reduction technique. (**a**) ODMR spectrum. (**b**) The same ODMR spectrum with digital LIA.

In our confocal microscope, fluctuations in laser intensity and mechanical vibrations degrade sensitivity measurements. To overcome these problems, we implement a digital form of Lock-in amplifier (LIA) which enhances the signal to noise ratio. We modulate the signal by modulating the MW field. Then, the output of the photon counter is demodulated in software. Figure (11a) shows an ODMR spectrum measured directly with continuous MW field. To generate such a spectrum, we usually have to average more than 100 times. Figure (11b) is the same ODMR spectrum in (a) generated with modulated MW field. This spectrum did not need averaging at all, but every point took one second of accumulation time. We utilized this digital LIA technique in generating data used in Figs (7) and (8).

To measure the magnetic sensitivity of our shallow layer of NVs, as in Fig. (8), we set the MW frequency at 2860 MHz, which approximately corresponds to one of the points of highest sensitivity as illustrated in Fig. (10). Then, we modulated the MW field at 1 kHz and measured the NVs’ fluorescence intensity for 1 second. To generate the error bars, we repeated the measurement ten times for each value of the magnetic field and calculated the standard deviation of the measurements. From these statistics we calculated a magnetic sensitivity of $8.6\,\mu T/\sqrt{Hz}$.

For NV-based magnetometers, the magnetic field is measured by measuring the brightness of the NVs’ fluorescence. The signal to noise ratio for this measurement scales with the square root of the brightness. So, assuming the NVs are distributed uniformly, brightness increases linearly with the number of NVs and so sensitivity of the measurement improves (the number goes down) with the square root of the number of NVs.

The $8.6\,\mu T/\sqrt{Hz}$ sensitivity was acquired with a confocal microscope. In this arrangement the integration area is the laser spot size. Our confocal microscope is diffraction limited and we use an oil immersion objective lens with 1.4 NA. So, we assume that the spot size diameter is around 300 nm. This corresponds to an area of $\pi \ast {(0.15)}^{2}=0.0707\,\mu {m}^{2}$. We get the magnetic sensitivity per unit area by multiplying $8.6\ast \sqrt{0.0707}$ = $2.29\,\mu T/\sqrt{Hz\cdot \mu {m}^{-2}}$. Similarly, assuming thickness of 3 nm, the sensitivity per unit volume is given by $2.29\ast \sqrt{0.003}$ = $125\,nT/\sqrt{Hz\cdot \mu {m}^{-3}}$.

### Depth estimation with FRET

Depth estimation via FRET is simple and easy to perform, however, it is not as informing as nuclear magnetic resonance (NMR) depth estimation techniques^36^. In Fig. (6) we show quenching of the NVs’ fluorescence when we put a layer of BHQ3 on the diamond’s surface. We attribute this quenching to FRET coupling between NVs and BHQ3 molecules. However, quenching can be caused by other effects.

**Figure 12:**
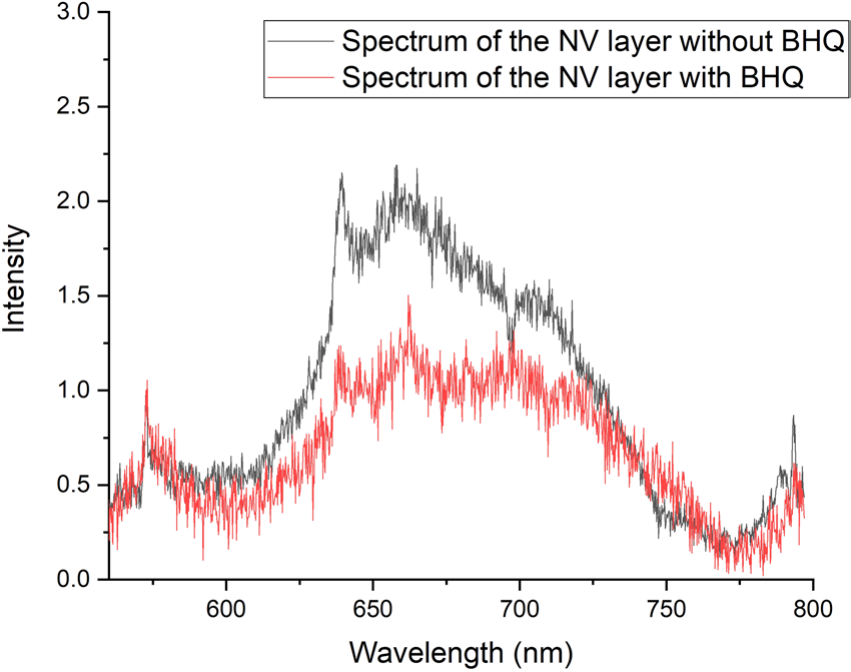
Spectra of shallow NV layers with (red) and without (black) BHQ quencher. Spectra are normalized with the diamond Raman line at 573 nm.

In Fig. (12) we show optical spectrum of our shallow NVs with and without the BHQ3 layer. We point out that the spectra of the NVs in both cases have the same profile and there is no visible trace of *NV*^0^ spectrum. Moreover, since both spectra are those of *NV*^−^, we know that brightness measured in Fig. (6a) is coming from the NV layer and not from contamination on the diamonds surface.

**Figure 13:**
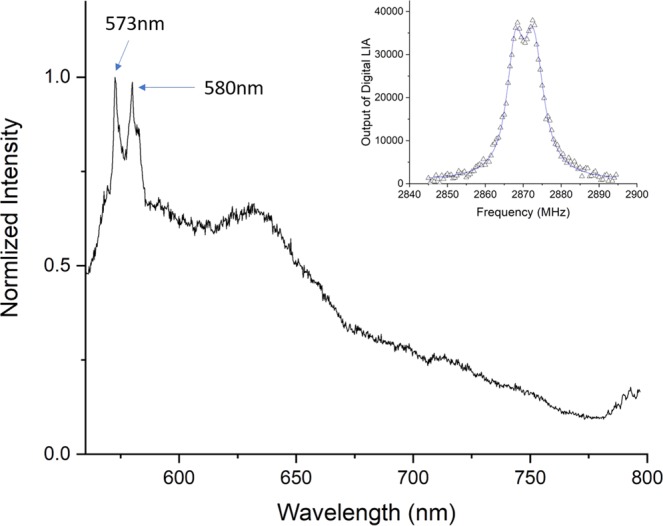
Spectrum of a thin layer of NVs grown with CVD. The layer is around 5 nm thick according to growth parameters. The peaks at 573 nm and 580 nm are the diamond Raman peak and the graphite peak respectively. To prove this layer indeed has *NV*^−^ in it, we generated an ODMR spectrum from this layer (inset).

**Figure 14:**
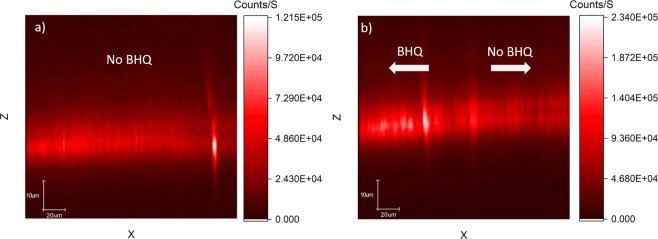
XZ confocal scans of the thin CVD layer in Fig. 13. (**a**) A confocal scan before adding BHQ3. (**b**) A confocal scan after adding BHQ3. In B the BHQ3 is to the left of the scan and the bright spots were not quenched. In general, there weren’t any signs of FRET quenching on this sample.

Furthermore, we attempted FRET coupling with two CVD grown shallow NV layers; a 5-nm thick layer, and 1-micron-thick layer, as estimated by growth parameters. The thin sample was generally dim and had some bright spots where the spectrum was a mixture on *NV*^0^, *NV*^−^, and graphite. Figure 13, shows the optical spectrum of the layer, where the peaks at 573 nm and 580 nm are the diamond Raman peak and the graphite G peak respectively. In the inset we add the ODMR spectrum of this layer as a proof it, indeed, contains *NV*^−^. In Fig. (14) we show confocal scans of this sample without (a) and with (b) BHQ3 layer on top. There was no clear effect of BHQ3 on this sample. In fig. b we can see that the BHQ layer could not quench the bright spots (to the left of the graph).

The thick sample had a generally uniform bright layer, which had an excellent *NV*^−^ spectrum. However, adding BHQ to the surface of the layer did not have any effect on the brightness nor the spectrum of the NV layer. All confocal scans of this sample showed a uniform layer without any signs of quenching. Even though, our data strongly suggests that the quenching is indeed due to FRET coupling, NMR depth estimation experiments might be necessary to assert this depth estimation claim.
